# 3D scaffolds of caprolactone/chitosan/polyvinyl alcohol/hydroxyapatite stabilized by physical bonds seeded with swine dental pulp stem cell for bone tissue engineering

**DOI:** 10.1007/s10856-022-06702-2

**Published:** 2022-12-09

**Authors:** V. A. Reyna-Urrutia, Miriam Estevez, A. M. González-González, R. Rosales-Ibáñez

**Affiliations:** 1 Tissue Engineering and Translational Medicine Laboratory, Iztacala School of Higher Studies, National Autonomous University of Mexico, Tenayuca-Chalmita S/N, Cuautepec Barrio Bajo, Gustavo A. Madero, Mexico, CP 07239 Mexico; 2 Center for Applied Physics and Advanced Technology, National Autonomous University of Mexico, Campus Juriquilla, Boulevard Juriquilla No. 3001, Querétaro, Juriquilla, CP 76230 Mexico

## Abstract

Bone Regeneration represents a clinical need, related to bone defects such as congenital anomalies, trauma with bone loss, and/or some pathologies such as cysts or tumors This is why a polymeric biomaterial that mimics the osteogenic composition and structure represents a high potential to face this problem. The method of obtaining these materials was first to prepare a stabilized hydrogel by means of physical bonds and then to make use of the lyophilization technique to obtain the 3D porous scaffolds with temperature conditions of −58 °C and pressure of 1 Pa for 16 h. The physicochemical and bioactive properties of the scaffolds were studied. FTIR and TGA results confirm the presence of the initial components in the 3d matrix of the scaffold. The scaffolds exhibited a morphology with pore size and interconnectivity that promote good cell viability. Together, the cell viability and proliferation test, Alamar Blue^TM^ and the differentiation test: alizarin staining, showed the ability of physically stabilized scaffolds to proliferate and differentiate swine dental pulp stem cell (DPSCs) followed by mineralization. Therefore, the Cs-PCL-PVA-HA scaffold stabilized by physical bonds has characteristics that suggest great utility for future complementary in vitro tests and in vivo studies on bone defects. Likewise, this biomaterial was enhanced with the addition of HA, providing a scaffold with osteoconductive properties necessary for good regeneration of bone tissue.

**Figure:**
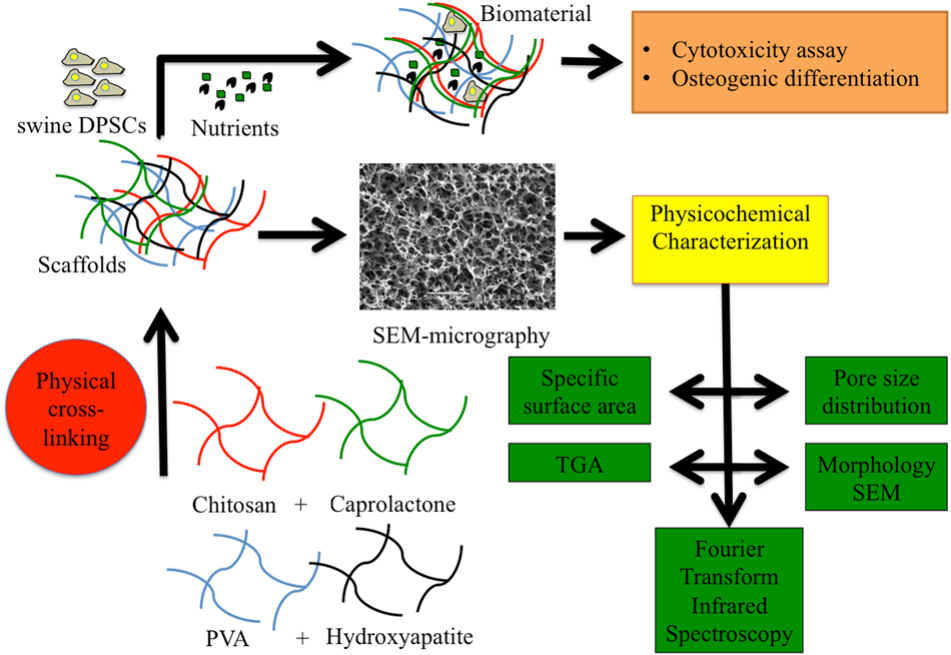
Graphical abstract

Graphical abstract

## Introduction

The craniofacial unit is a truly complex and damage to these structures, even minimal, usually leads to a deformity that can cause side effects in the patient. Advances in surgical techniques and the implementation of bone grafting have significantly improved the function and cosmetic restoration of craniofacial structures due to accidents (trauma) or disease. The regeneration of oral and craniofacial tissues currently represents a challenge in medicine and for this the synthesis of basic, clinical and engineering science is required [1].

If we look at the world statistics we find in the United States alone, more than 30,000 patients who undergo craniofacial resection surgery per year. Therefore, this expansion of figures provides support to justify the investigation of regenerative medicine resources to face this problem [2].

Now, if we take into account the high rate of patients with different degrees of bone involvement, it is feasible to see the alternatives to improve this type of problem in some way. This is why tissue engineering constitutes a broad field of study and practical application with a high growth potential, which is why this research designs and characterizes a material with properties for bone regeneration that can be used in bone disorders of the patients who present it.

There are different materials used in the elaboration of scaffolds for tissue engineering; some are of synthetic origin and others of natural origin, chitosan (Cs) being one of the most important of the latter group. This is a polysaccharide that is commonly obtained by extensive deacetylation of the chitin of some crustaceans [3]. Cs is a biopolymer that can be used in various biomedical applications due to its low toxicity and its bioactive properties (hemostatic, antimicrobial activity, biocompatibility, etc.). Furthermore, it is commercially available, at a relatively low cost to produce biomaterials in the pharmaceutical and medical fields [4].

Another material for designing tissue supports is poly(ε-caprolactone) or also known as PCL, it can mimic the matrix of the bone tissue to be repaired, offering properties that favor its use in this application and its manufacture as 3D scaffolds, generating a structure with porosity in which a feasible environment can be created to generate new bone tissue [5, 6]. An important feature of PCL is that it is a polymer that is soluble in a wide range of organic solvents, which makes it a promising material for research, as it could be mixed with a variety of polymers to design composite biomaterials [7].

Among the synthetic biomaterials, poly(vinyl alcohol) (PVA) has great potential for the production of scaffolds due to its high hydrophilicity, permeability, biodegradability, biocompatibility, flexibility, and ability to mix with other biopolymers. Furthermore, PVA is an excellent material for the production of foams and emulsions [8].

Another material is hydroxyapatite (HA), it is a ceramic and crystalline compound with a hexagonal network and has a specific formula [Ca_10_(PO_4_)_6_(OH)_2_], being the main constituent mineral of teeth and bones [Ca_10_(PO_4_)_6_(OH)_2_] [9]. Regarding this characteristic, it is biocompatible and does not produce an inflammatory response [10, 11]. HA is found in nature, forming porous structures that vary depending on the bone site from which it is extracted, for example, trabecular bone has 65% porosity and 100–200 µm in diameter, which allows it to have osteoconductive properties [12]. Another important characteristic is its slow resorption, which usually keeps the material in its initial state for 2-3 years after implantation. This allows slow growth of bone tissue with cell proliferation within the material [2, 13]. HA also shows very good mechanical properties with a compressive strength of up to 160 MPa, with applications in small areas of bone under low load conditions [12, 14, 15].

Until now, the scaffolds that make use of these polymers have designed their matrices through a variety of techniques such as electrospinning or lyophilization, and in their methodologies they use crosslinking agents to improve their mechanical properties and chemical stability [16, 17]; however, these crosslinking agents affect the biological properties of the material, which is a characteristic of great importance in the biocompatibility tests of the scaffold. In some studies, better biocompatibility (scaffold has non-cytotoxic properties and it doesn´t have any functional group that can damage cells) and cell viability have been found in supports manufactured by the lyophilization process compared to electrospinning [18]. Despite the above, during the freezing process of the support, the Cs chains are affected, forming agglomerations that reduce the availability of functional groups (-NH_2_ group) related to the biocompatibility of the Cs molecule [19]. To reduce this effect, different cross-linking agents have been used, such as glutaraldehyde, genipin, oxaldehyde, etc., although these compounds have been considered cytotoxic or of unknown biocompatibility [20]. This is why it is intended to study an alternative method of forming a physical gel of Cs-PCL-PVA-HA without using cytotoxic chemical compounds as cross-linking agents, in order not to affect the biocompatibility of the material, as well as leaving available the amino groups in the Cs molecule, which are generally involved in the crosslinking reaction; In this way, chemical crosslinking with unknown bioactivity for the stability of scaffolds would be avoided, mimicking the extracellular structure of tissues. Using ammonium hydroxide, generating physical entanglements instead of chemical crosslinking reactions, the hydrogel is then lyophilized to obtain 3D scaffolds and thus evaluate the osteogenic lineage induction properties.

## Materials and methods

### Materials

Chitosan (Cs) with catalog No. 448869 y 419419, polycaprolactone (PCL) with catalog No. 440752, poly(vinyl alcohol) (PVA) with catalog No. 363146 and hydroxyapatite with catalog No. 289396 were used in the preparation of the scaffolds. Ammonium hydroxide (HA, 28% NH_3_ in H_2_O, ≥ 99.99% trace metals basis, No. 338818), dichloromethane (DC, high-purity grade ≥99.8%, No. C1470) and glacial acetic acid (AA, ≥ 99.5% purity No. A6283) were used to make the hydrogels. All solvents and reagents are Sigma-Aldrich brand reagent grade (Toluca, Mexico).

### Preparation of solutions

50/50% of each batch of chitosan was mixed and a 2.5% (w/w) chitosan solution was prepared by dissolving it in 0.2 M glacial acetic acid at room temperature and stirring for 24 h. Then, the solution was degassed.

A polycaprolactone solution at 5% (w/w) was prepared, dissolving in dichloromethane room temperature and stirring overnight. Then, the solution was degassed.

A poly (vinyl alcohol) solution at 5% (w/w) was prepared, dissolving in dichloromethane at room temperature and stirring overnight. Then, the solution was degassed.

### Composite scaffolds

Cs materials (100%) and mixtures of Cs/PVA (80/20), Cs/PCL (80/20), Cs/PVA/PCL (80/10/10) and Cs/PVA/PCL/HA were made (79/10/10/01), ratio (p/p) respectively, with constant stirring for 24 h at room temperature. The polymer blend was aliquoted and placed into a sealed container in the presence of ammonium hydroxide. Physical crosslinking (gelation) was induced by ammonia diffusion for 24 h [21]. Subsequently, the hydrogels were washed with distilled water to remove the ammonium acetate (CH3COONH4) and the rest of the ammonium hydroxide that remained in the hydrogels (NH4OH), until a pH of 7 is obtained, these residues can have an influence on the biocompatibility of biomaterials. To obtain the 3D constructs, the hydrogels were placed in a FREEZE DRYER model SCIENTZ-10N at −58 °C, and a pressure of 1 Pa for 16 h [22].

### Physicochemical characterization of 3D scaffolds

#### Scanning electron microscope (SEM)

The morphology of the structure of the porous constructs was observed by scanning electron microscopy (SEM), using Hitachi SU8230 equipment at 1.0 kV. The scaffolds were placed on an aluminum tape and covered with a layer of gold using an LLC model to high vacuum Desk II.

#### Specific surface area

The analysis was carried out by nitrogen adsorption at 77 K to determine the specific surface area of the scaffolds, using a Quentachome NOVA 2200e Instruments. The samples were degassed for 12 h, at 95 °C, before the measurements in order to eliminate the air and moisture present in the samples. The Brunauer-Emmett-Teller (BET) model was applied to fit the isotherms and calculate the specific surface area of the analyzed materials [23].

#### Fourier transform infrared spectroscopy (FTIR)

The spectra of the biomaterials were obtained using a Perkin Elmer Spectrum Two FTIR spectrometer. Spectra were analyzed in the wavenumber range of 4000–650 cm^−1^ with a resolution of 4 cm^−1^ and a ratio of 100 scans.

#### Thermal gravimetric analysis (TGA)

The thermogravimetric study was carried out in a Mettler Toledo TGA/DSC 2+ thermal analyzer, with a nitrogen atmosphere. Constructs were measured between 25 °C and 600 °C with a heating ramp of 20 °C/min.

### Biological characterization of 3D scaffolds

#### Sample sterilization

The samples were sterilized by exposure to UV radiation for 20 min (on each side) using a laminar flow hood (Telstar®, Bio II advance, type Class II).

#### Cytotoxicity assay

The cell viability assay was analyzed using the colorimetric Alamar blue™ (Invitrogen) Cellular Metabolic Assay. The biomaterials were placed in 96-well culture plates and then, swinw dental pulp stem cells (DPSCs) (8 × 10^6^) were seeded on the scaffolds and similarly seeded in 96-well culture plates but without scaffolds as a control group, cells were grown in a Dulbecco’s Modified Eagle Medium (DMEM) low-glucose (Bio-west, Mexico), added with 10% Fetal Bovine Serum (FBS) (Bio-west, Mexico) and 1% penicillin-streptomycin (Sigma-Aldrich). The incubation conditions were at 37 °C, in an atmosphere with 5% CO_2_ and 95% humidity. At times of 3, 7 and 10 days, the medium was removed, 90 µL of fresh medium and 10 µL of Alamar blue™ were added, then the cells were incubated for 4 h under the same conditions mentioned. To end the assay, cell viability was read at a wavelength of 630 and 492 nm in a microplate reader (PKL PPC 142, Pokler Italy). To eliminate the experimental error, three repetitions were made in each of the treatments used in the analysis.

#### Osteogenic differentiation of swine DPSCs on a scaffold

A cell density of 2 × 10^6^ of swine DPSCs was first plated in a 96-well plate. The biomaterials were cultured with low-glucose DMEM (Bio-west, Mexico), adding 10% Fetal Bovine Serum (FBS) (Bio-west, Mexico) and 1% penicillin-streptomycin (Sigma Aldrich), and incubated at 37 °C, 5% CO_2_ (BINDER 13-16721 incubator, Germany) with humidity for 48 h. After the elapsed time, the culture medium was removed and new medium was added. This medium was changed twice a week for the duration of the 28-day trial. Swine DPSCs seeded on 3D biomaterials were cultured with MesenCult™ osteogenic differentiation medium (Human of MesenCult™ osteogenic, Stem Cells Technologies, Cambridge, MA, USA), in the same way medium changes were made twice a week during the 28 days of the induction assay, then Alizarin Red Staining was performed.

#### Alizarin Red Staining (ARS) activity

The principle of alizarin red S, is to selectively color calcium deposits and has been used in recent years to study calcium-rich deposits formed in cell cultures [24]. After 28 days of induction with MesenCult™ osteogenic differentiation medium, calcium-rich deposits produced by swine DPSCs on scaffolds (constructs) was assessed by Alizarin Red S staining (Sigma Aldrich, USA). For this purpose, constructs were fixed with 4% neutral formalin for 5 min, washed three times with PBS, and then washed with distilled water to remove any salt residues. The constructs were stained with 2% Alizarin red S (pH 4.2), so that it covered the entire surface of the constructs. After 1 h of incubation at room temperature, the ARS excess was washed with distilled water. To analyzed and capture the images of calcium-rich deposits, we used a Leica DM IL LED inverted light-field phase contrast optical microscope.

### Statistical analysis

Data analysis was expressed as a mean ± standard deviation. Statistical analysis was using a one-way analysis of variance (ANOVA) followed by Tukey’s test. For significant differences, a value of *p* < 0.05 was taken. SEM micrographs were used to obtain the pore size distribution with 50 measurements from five different fields using Adobe Ps© 1990–2021 software, then histograms were made for each scaffold in Microsoft Excel© software.

## Results

### Characterization of 3D scaffolds

#### Scaffolds morphology

**Fig. 1:**
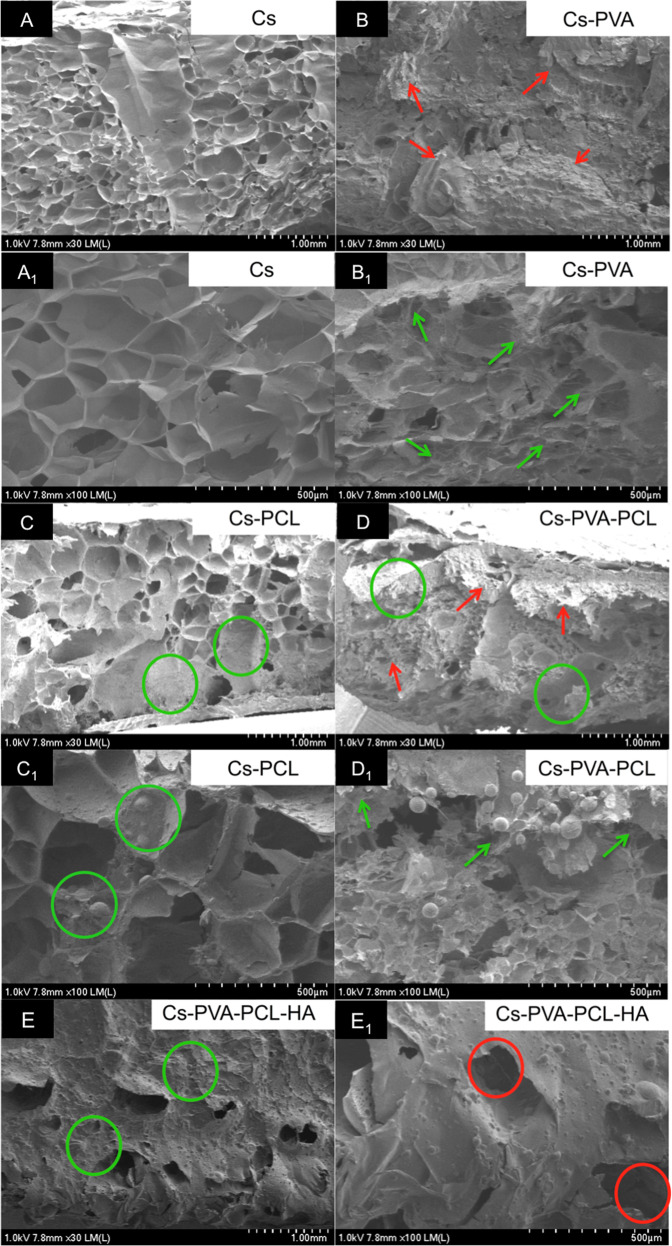
SEM micrographs of scaffolds obtained at ×30 (**A**–**E**) and ×100 (**A**_**1**_–**E**_**1**_) magnification, respectively. The area marked in green circles (1C, 1C_1_ 1D and 1E), can be correspondents to PCL because the scaffold without PCL (image 1A and 1A_1_) it does not have a morphology with rounded parts. This same behavior is observed in previous research with Cs-PCL scaffolds. Images 1-B and 1-B_1_ show a structural morphology with cobweb-shaped aggregates, changes that were shown when PVA was added to the scaffold (green arrows, image 1-B_1_) and not in a rounded shape

Figure 1 shows SEM micrographs of scaffolds obtained at ×30 (A-E) and ×100 (A_1_-E_1_) magnification, respectively. As it can be seen, Cs exhibit the most homogeneous morphology of all the prepared scaffolds. In contrast, the SEM micrographs for the Cs-PVA-PCL and Cs-PVA-PCL-HA sample showed a heterogeneous morphology, which includes areas where the PCL component may be in a matrix of the porous scaffold (green circle in Fig. 1C, 1C_1_, 1D and 1E), their results show morphologies similar to ours when adding PCL to the scaffold [25, 26]. Similarly, a certain similarity can be seen in the Cs-PVA and Cs-PVA-PCL scaffolds, where there are some areas (see red arrows in Fig. 1B and D) in which morphology in the form of clusters prevails. On the other hand, the addition of PVA to the matrices (see green arrows in Fig. 1B_1_ and D_1_) produced materials with a very different morphology with the presence of entanglements. This indicates that PVA has a strong influence on chitosan polymer chains, which produce three-dimensional structures with this peculiarity. The presence of this type of entanglement-shaped structures was less in the Cs-PVA-PCL-HA scaffold. Finally, although the Cs-PVA-PCL-HA scaffold showed the largest morphology and heterogeneity, it also presented interconnectivity (see red circle in Fig. 1E1) of pores in the three-dimensional structure.

#### Pore size distribution and specific surface area

**Fig. 2:**
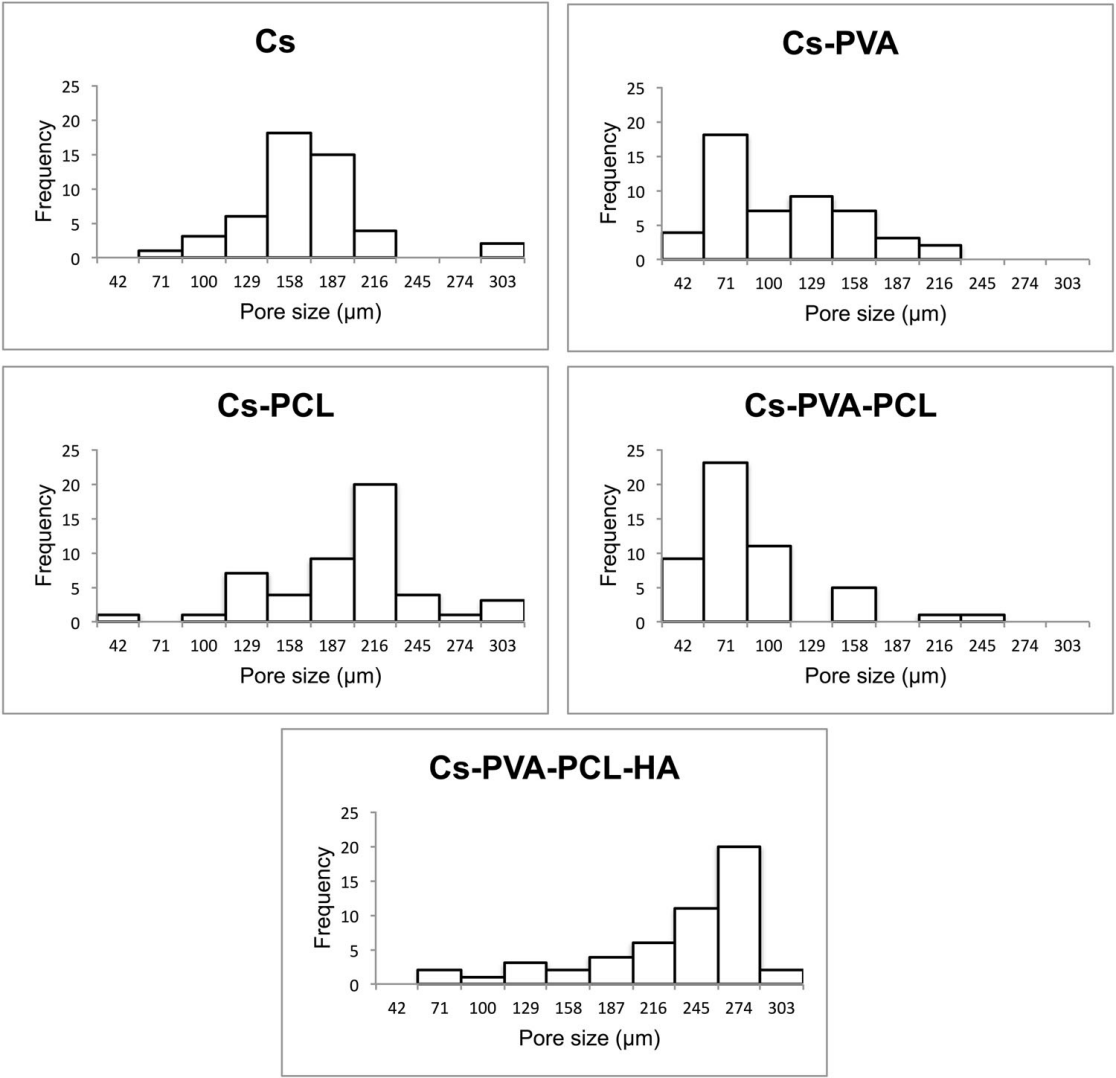
Pore size distribution of 3D-scaffolds obtained by freeze-drying from hydrogels

Figure 2 shows the pore size distribution of the scaffolds prepared in this investigation. In general, all samples were found to have a unimodal distribution. It is noteworthy that the surface of the Cs samples shows a symmetric distribution with values in a greater number of repetitions in the range of 144–172 μm that predominate in relation to the rest of the treatments where asymmetric distributions are observed. In this sense, the Cs-PVA and Cs-PVA-PCL samples exhibited a right-skewed distribution with values for both in a higher repetitive number in the range of 57–85 μm, while the Cs-PCL and Cs-PVA-PCL-HA tend to have a leftward distribution with repetitive values in the range of 203–230 and 260–268 μm, respectively.

The specific surface area (SSA) of the scaffolds is shown in Table 1. The material that presented a higher specific surface area was Cs-PVA and it has a relationship with respect to all the other mixtures. Where the incorporation of PVA gives the material an increase in the S_BET_ value, the opposite occurs when working with PCL where the materials suffer a decrease in surface area. However, the Cs-PVA-PCL-HA scaffold does not suffer a drastic drop in values, thanks to the presence of PVA in the geometry of the material matrix.

**Table 1:** Average pore size (T_p_) and specific surface area (SSA) of 3D-materials

Material	T_p_ (μm)*	SSA (cm^2^/g)
Cs	163.5 ± 42.5	283.7 ± 4.2
Cs-PVA	107.0 ± 44.2	388.6 ± 6.7
Cs-PCL	197.1 ± 50.9	6.3 ± 0.26
Cs-PVA-PCL	88.7 ± 41.4	60.4 ± 1.3
Cs-PVA-PCL-HA	222.7 ± 62.7	132.6 ± 62.7

*Average value from 50 measurements

#### Fourier Transform Infrared Spectroscopy (FTIR)

**Fig. 3:**
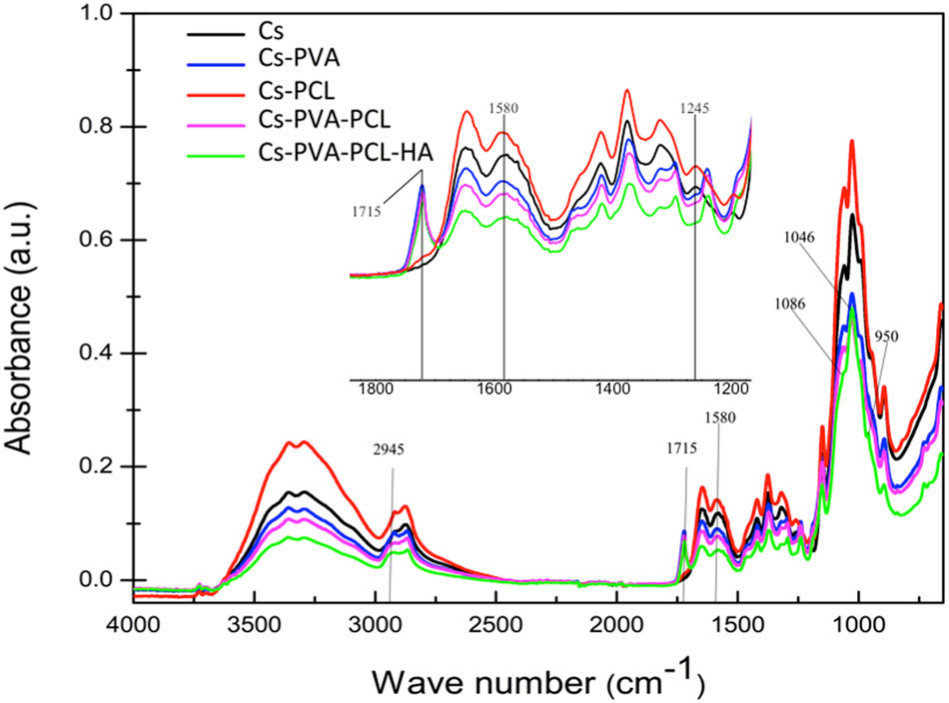
FTIR spectra of biomaterials

Figure 3 presents the infrared spectra of the scaffolds made in this investigation. All the spectra were similar to each other, showing the characteristic absorption bands of Cs; in the wave number range of 3500–3300 cm^−1^, a broad and intense band was observed, assigning the stretching vibrations O-H and N-H. In addition, bands were observed at 1653 cm^−1^, which is associated with the C = O stretch of amide I and, at 1580 cm^−1^, related to the deformation of amide II [27–29]. Peaks were also detected at 2923 and 2880 cm^−1^, associated with methylene groups and, finally, in the range of 1200–1000 cm^−1^, characteristic signals of the saccharide structure [30].

The absorption band at 1580 cm^−1^, shown by the FTIR spectrum of the Cs material, was shifted to lower wavenumbers in the Cs-PVA, Cs-PCL, Cs-PVA-PCL and Cs-PVA-PCL-HA. This fact confirms interaction between Cs with the components of the mixture. The absorption band of the Cs-PVA, Cs-PVA-PCL and Cs-PVA-PCL-HA scaffolds show a peak at 1715 cm^−1^ which is characteristic of the C = O group and confirms the presence of PVA in these scaffolds [31], in the same way it is observed that the Cs band at 2923 cm^−1^ shifted to 2945, which also indicates the presence of PVA in said scaffolds [32]. The FTIR of the Cs-PCL scaffold shows a band at 1245 cm^−1^ that is characteristic of the C = O of the PCL, this intensity is diminished when it is determined for the Cs scaffold (100%) [33, 34].

On the other hand, in the Cs-PV-PCL-HA scaffold around 1000 cm^−1^, is the most important signal of hydroxyapatite is found, the antisymmetric vibration of the phosphate group ν_3_^as^ (PO_4_^3-^), which is identified by the presence of a doublet with defined maxima around 1087 and 1046 cm^−1^. In the same way, the absorption band appears at 950 cm^−1^ which is characteristic of the symmetric vibration of ν^s^_1_ (PO_4_^3-^) of this polymer [35–37].

#### Thermal gravimetric analysis (TGA)

**Fig. 4:**
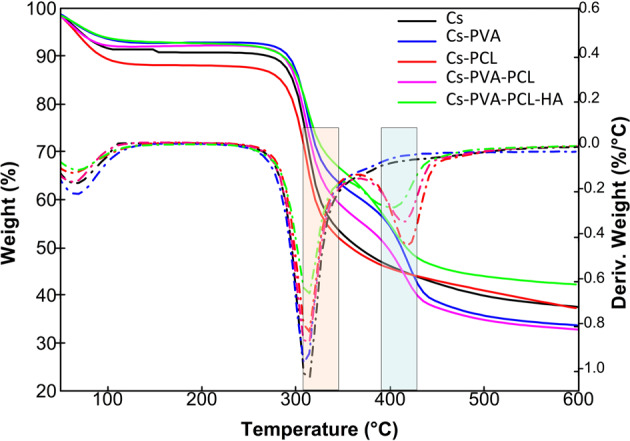
Thermogravimetric analysis of biomaterials

Figure 4 presents the TGA analyzes for the samples of all the scaffolds prepared in this work. It can be seen that the Cs-PVA, Cs-PCL, Cs-PVA-PCL and Cs-PVA-PCL-HA scaffolds show thermal stability superior to that of Cs, which is indicated at 309 °C (see orange zone) and is characteristic of the use of another component in the scaffold matrix mix [38].

### Biological characterization of 3D scaffolds

#### Cytotoxicity assay

**Fig. 5:**
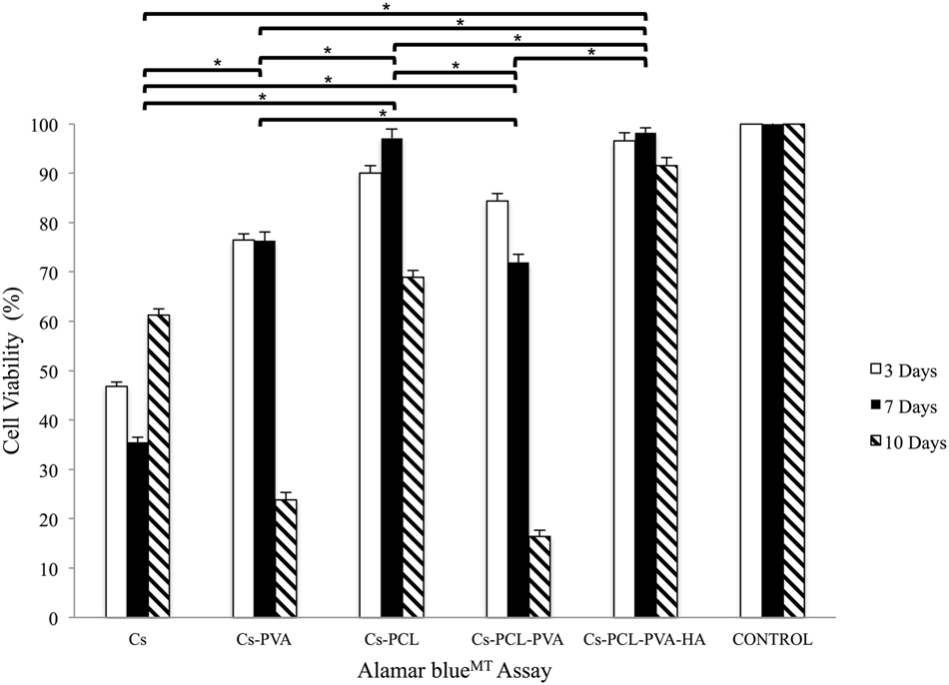
Cell viability (Alamar blue^TM^ cell metabolic assay) in control and in 3D scaffolds groups. ANOVA one way, *P* < 0.05, Tukey’s test. * Indicates statistically significant difference between groups

There are many methods to evaluate the cytotoxic effect of biomaterials on cultured cells by monitoring non-specific alterations in basic cell function such as mitochondria, plasma membrane integrity, etc. [39]. One of them is the cell metabolic Alamar blue^TM^, where the results of cell proliferation and viability are shown in Fig. 5, it was analyzed by ANOVA one way, *P* < 0.05, Tukey’s test. The Cs-PVA and Cs-PCL-PVA scaffolds showed a decreasing proliferation throughout the study time, so the concentration or the presence of the PVA polymer in the scaffold matrix is being involved in the results.

#### Osteogenic differentiation of swine DPSCs on a scaffold

**Fig. 6:**
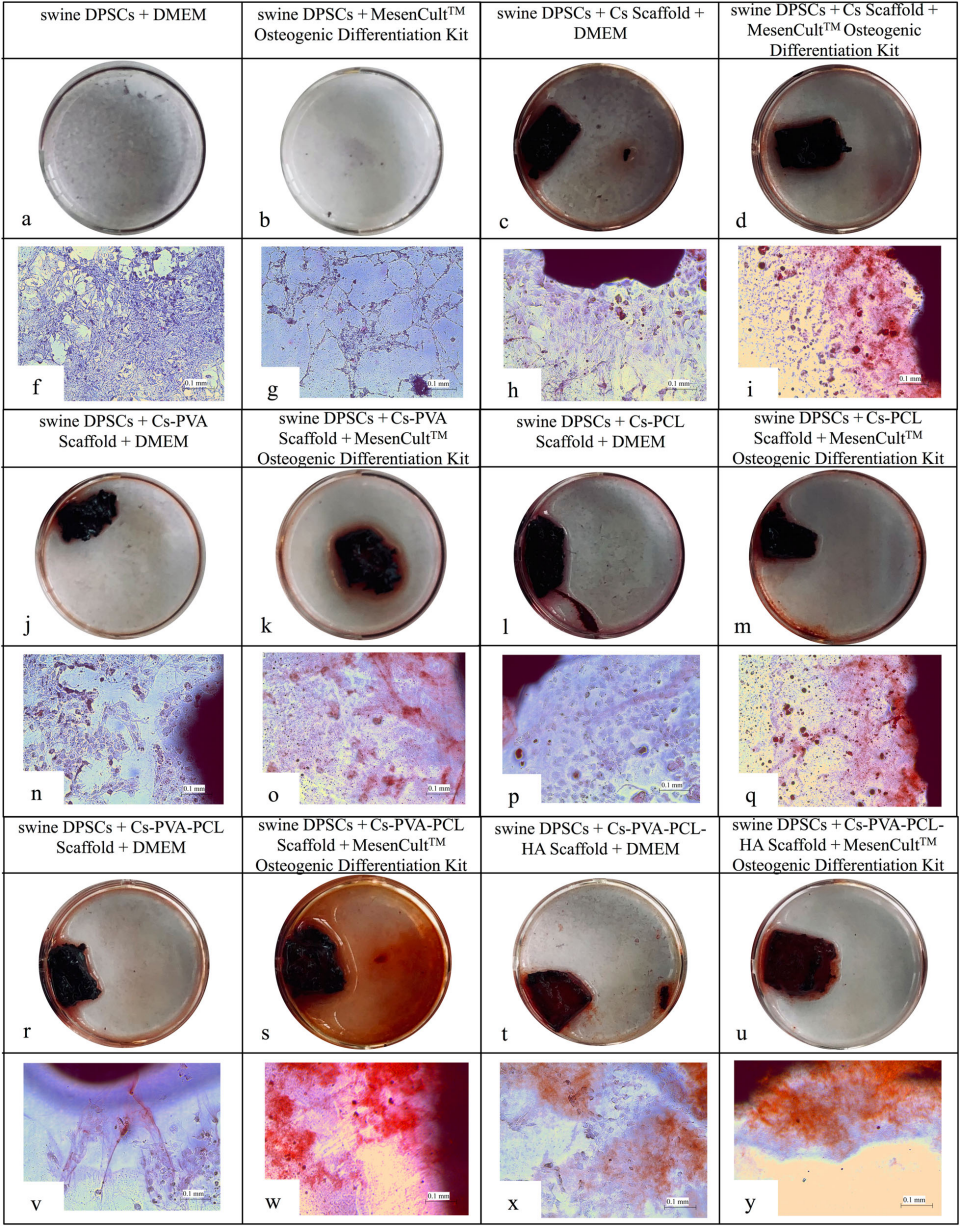
Representative images of the mineral deposition in swine DPSCs on day 28 by Alizarin red S staining (**a**–**d**, **j**–**m** and **r**–**u**) and microscopy images (**f**–**i**, **n**–**q** and **v**–**y**) at the bottom 10x

The swine DPSCs seeded in the different 3D scaffolds of this investigation are shown in Fig. 6, making use of the MesenCult^TM^ Osteogenic Differentiation Kit. The results show a greater differentiation and mineralization of calcium deposits when the osteogenic MesenCult^TM^ kit is used than when using only DMEM as the medium, but a greater deposition of calcium is also observed in this rather than in the control group.

## Discussion

One of the most promising characteristics of Cs for its use in the manufacture of scaffolds is its excellent results, which gives the formation of an open pore microstructure with a high degree of interconnectivity [40], a characteristic that was presented in the materials obtained from this investigation (see Fig. 1).

Another peculiarity is the pore size of chitosan scaffolds that are in the range of 50–300 μm, it is important that the pores have an adequately large size so that the cell can penetrate and present interconnectivity to facilitate the exchange of waste and nutrients by the cells inside the elaborated construct [29, 41], allow the interaction of the polymer with the cell and the tissue for cell growth and differentiation for the formation of three-dimensional tissue with a formation of new bone [42, 43], these values of size of pore are close to those obtained in this research (see Fig. 2), which makes the use of these scaffolds promising.

The SSA of the obtained scaffolds was, in general, similar to the values reported for other three-dimensional chitosan-based scaffolds, which were also freeze-dried. For example, there are studies in which surface areas of 200 cm^2^/g were found for Cs scaffolds frozen at −80 °C [44]. This similarity could be due to the fact that our hydrogels were not subjected to a prior freezing process, but rather were placed directly in the cooling system included in the lyophilization equipment. In this way, the hydrogels were subjected to rapid a freezing process, which allows a better preservation of the gel structure, as reported by Robitzer et al. [23]. In this sense, it is well known that freezing prior to lyophilization helps to improve the morphological properties and the formation of ice crystals, but it should also be considered that a faster freezing (produced by pressure reduction) produces a speed of faster freezing that causes rapid ice nucleation, giving rise to smaller crystals [45]. This could explain the values of SSA in the scaffolds obtained in this work (see Table 1).

The TGA analysis, the scaffolds of Cs-PCL, Cs-PVA-PCL and Cs-PVA-PCL-HA scaffolds present a second peak in the decomposition weight derivative (marked in the blue area, see Fig. 4), which is characteristic of the use of PCL in the scaffolding mix [46]. Hydroxyapatite had no weight loss in the temperature range investigated. It was shown that the presence of hydroxyapatite displaces the first degradation profile at higher temperatures (219 °C), which indicates a better thermal stability of the chitosan matrix in the presence of inorganic compounds that probably hinder the thermo-oxidation of the organic matrix [47]. The residual weight of the Cs-PVA-PCL-HA scaffold showed at a temperature of 600 °C a weight loss of 43–44%, a higher value with respect to the other scaffolds elaborated in the investigation, indicating the presence of HA in chitosan-based compounds.

In the cell viability assay (see Fig. 5), there is a decrease in the number of cells and this is due to the difficulty of the cells to adhere to the scaffold with a highly hydrophilic nature of PVA [48, 49], so it could be observed that the content of PVA in the mixture had a direct effect on cell viability. Similarly, Sánchez-Cardona *et al*., [32], had low cell viability when they increased the concentration of PVA in the scaffold that they designed. On the other hand, a significant increase was shown from 3 to 10 days for the Cs, Cs-PCL and Cs-PCL-PVA-HA scaffolds. Where the latter presented the best performance throughout the trial with non-significant values with respect to the Control, so it had a very similar behavior [32]. Result that was positive thanks to the use of HA in the design of the tissue construct. Said result is compared with that of Oliveira et al. [50] where viability results are obtained that were better when they used HA in the three-dimensional matrix of the scaffold. Therefore, these results have potential to be applied in bone tissue engineering [50].

As described in the literature, during early osteogenic induction, stem cells continue to proliferate and migrate [51, 52]. Some cells have adhered to the scaffolds, but other cells have migrated from the scaffold to the bottom of the well plates, as can be seen in Fig. 6 (h,i n-q, v-y) [53]. Later, some of these cells have begun to secrete extracellular matrix in the lower part of the well plate and also in the scaffolds, which can be seen in Fig. 6-t and 6-x, which corresponds to the Cs-PVA-PCL-HA scaffold. The above is only making use of the DMEM medium; this is of great importance because the use of osteogenic MesenCult^TM^ kit was not necessary, so adding HA in the design of the tissue construct presents osteoconductive activity.

## Conclusions

The scaffolds made in this research by forming a gel (physically stabilized) and subsequently dried using the lyophilization technique presented morphologies with pore size within the characteristics necessary for cell proliferation. The scaffolds presented a specific surface area which could help the bioactivity of the functional groups of the polymers that make up the designed matrices. The FTIR and TGA results confirm the presence of the polymeric components in the three-dimensional matrices. The Cs, Cs-PCL and Cs-PCL-PVA-HA scaffolds had no cytotoxic effect at 10 days evaluated with the Alamar blue^TM^ cellular metabolic assay. The scaffolds support the adhesion, viability and proliferation of swine PSCs and have a slight effect on mineralization capacity in Cs-PCL-PVA-HA scaffolds.
